# Cocaine use disorder in females is associated with altered social decision-making: a study with the prisoner’s dilemma and the ultimatum game

**DOI:** 10.1186/s12888-019-2198-0

**Published:** 2019-07-05

**Authors:** Thiago Wendt Viola, João Paulo Otolia Niederauer, Bruno Kluwe-Schiavon, Breno Sanvicente-Vieira, Rodrigo Grassi-Oliveira

**Affiliations:** 1 Pontifical Catholic University of Rio Grande do Sul (PUCRS), Developmental Cognitive Neuroscience Lab, Avenida Ipiranga 6690 – Prédio 63, Jardim Botânico, Porto Alegre, RS Brazil; 2 Experimental and Clinical Pharmacopsychology Laboratory Department of Psychiatry Psychotherapy and Psychosomatics Psychiatric Hospital, University of Zurich, Zürich, Switzerland

**Keywords:** Cocaine, Cocaine-related disorders, Decision making, Cognition

## Abstract

**Background:**

Chronic cocaine use is associated with cognitive deficits, including poor performance on neuropsychological tasks of memory, executive functions, theory of mind and decision-making. However, the relationship between cocaine use disorder and social decision-making remains unclear. This is particularly relevant given the fact that many cocaine abusers present impairments in social functioning. In this sense, game theory paradigms have been helping to comprehend the behavior of psychiatric patients when they directly engage in social situations, which may better approximate many of their real-life choices.

**Methods:**

The present study investigated social decision-making in individuals with or without cocaine use disorder, examining their behavior in the Prisoner’s Dilemma and in the Ultimatum Game. Thus, 129 females diagnosed with cocaine use disorder and 55 females with no history of substance abuse were recruited and performed both social decision-making tasks. Additional assessments included information about demographics, patterns of substance consumption and executive function performance.

**Results:**

Females with cocaine use disorder opted more often to not defect in the Prisoner’s Dilemma, while in the Ultimatum Game they frequently chose to accept the first and unfair offer as responders. These effects were more pronounced within females with long-term history of cocaine use. Associations between cocaine use disorder and altered social decision-making were independent from demographic and executive function variables.

**Conclusions:**

The influence of cocaine use disorder on social decision-making was detected in both game paradigms, resulting in more cooperative behavior in the Prisoner’s Dilemma and higher acceptance rate of unfair offers in the Ultimatum Game. Further studies should focus on investigating these associations to shed light on the putative biopsychosocial factors underlying the observed effects.

## Background

Human societies are built on interpersonal and group relationships. The ability to interact in complex social environments depends on a set of affective and cognitive processes, including the necessity of making judgments and taking decisions that guide our own behavior and that influence the others [1]. The term ‘decision-making’ refers to this specific brain function of selecting a course of action from multiple alternatives [2]. The psychological processes (e.g. trust, cooperation, fairness, altruism, etc.) underlying such decisions in social contexts are comprehensively conceptualized as “social decision-making”, and these have been initially investigated in the field of behavioral economics [3].

In recent years, the incorporation of these studies by the field of behavioral neuroscience resulted in several advances in the understanding of psychopathology associated with social functioning impairments [4, 5]. Particularly, game theory paradigms have been helping to comprehend the behavior of psychiatric patients when they directly engage in social situations, which may better approximate many of their real-life choices [1]. Such experimental paradigms were developed considering that participants must allocate specific resources between themselves and others taking into account predefined rules [1]. The Prisoner’s Dilemma (PD) and the Ultimatum Game (UG) are widely used paradigms in experimental psychology, and they test the outcomes of “cooperating” versus “defecting” in complex social situations [6, 7]. The objective of the dilemma is to minimize prison sentence (i.e. avoiding a negative outcome), while the goal of the UG is to maximize reward gains (e.g. money, chocolates), by choosing the best option in the delivered scenario. However, both paradigms involve the decisions of at least two players, for instance a “proposer” and a “responder”, resulting in a situation where participant’s actions may affect the other player options and decision [8]. Therefore, emerging evidence suggest that different mental disorders, such as Schizophrenia [9] and Autism Spectrum Disorder [10] are implicated with decision-making alterations recognized during social adaptation, measured by the PD or the UG. Despite that, the relationship between substance use disorders and social decision-making remains unclear [11].

Cocaine Use Disorder (CUD) is associated with social isolation and a wide variety of breaches of social rules [12]. Studies have shown that cocaine dependents have frequent issues in managing social and interpersonal problems (e.g., criminal involvement, difficulties in keeping a job, and family conflicts) [13, 14]. Moreover, with the progression of chronic cocaine use, individuals may present cognitive deficits, including poor performance on neuropsychological tasks of memory, executive functions, theory of mind (i.e. a major domain of social cognition) and decision-making domains [15–18]. These cognitive alterations are associated with lack of adherence to treatment, as well as with higher relapse rates and greater difficulties to adapt to demands of social relationships [19–21]. Furthermore, gender represents an additional contributor to differences in clinical outcomes among individuals with CUD [22, 23]. Although CUD prevalence is higher in men, recent data have showed higher drug use severity, more problems in domains related to childcare issues, criminal involvement, work-related problems and social support impairments among women [24]. Moreover, women who are drug dependents are more sensitive to the effects of interpersonal problems on craving and relapse than are men [25, 26].

Because game theory paradigms could help in the understanding of the psychological processes of trustiness, fairness and cooperative behavior among cocaine dependents [27], we set out to explore social decision-making using both the PD and the UG paradigms in a sample of females with CUD. We aimed to investigate whether women with CUD are more prone to cooperate or to defect in the PD compared to women without a history of substance abuse. In addition, we investigated if women with CUD are more prone to make fair or unfair choices in the UG. Finally, because data suggest that women present higher drug use severity than men, we explored whether the effects of CUD on social decision-making could be even more pronounced among those participants with long-term history of cocaine use.

## Methods

### Participants

The sample of this study consisted of females with (*n* = 129; cases) and without CUD (*n* = 55; controls). Participants with CUD were recruited within the same inpatient detoxification treatment facility for female drug and alcohol abusers in Porto Alegre, Brazil. Non-substance abusers were recruited by convenience in the same city. All included participants gave written consent to participate in this study and the study was approved in the ethics committee of PUCRS. For the clinical sample, the inclusion criteria were as follows: females aged 18–50 years, a diagnosis of CUD – physiological dependence of snorted or smoked cocaine (crack), a minimum of four years of formal education and an absence of co-morbid psychotic syndromes. Diagnoses of CUD were based on clinical and structured interviews following the DSM-IV (Diagnostic and Statistical Manual of Mental Disorders, 4th edition) criteria. For non-substance abusers, the inclusion criteria were as follows: females aged 18–50 years, no history of drug abuse, a minimum of four years of formal education and an absence of co-morbid psychotic syndromes. Participants were excluded if either severe cognitive deficits that resulted in an altered state of consciousness or agitation were observed during the experiments.

### Sociodemographic and clinical characteristics

The following data was acquired through a semi-structured interview: age, ethnicity, income, years of formal education, number of hospitalizations and if the participant has ever been in prison. Regarding the pattern of substance consumption, participants were inquired about the age of drug experimentation (e.g. alcohol, tobacco, cannabis and cocaine), and the number of days of substance use. The intensity of craving to consume chocolate was also inquired in a single question, in which participants rated the craving for chocolate in the moment of the experiment in a scale going from 0 (without any craving for chocolate) to 10 (the most sensation of craving for chocolate).

### Executive functioning

Given that some studies suggested that social decision-making consists in a specific cognitive domain, apart from other executive function abilities [28, 29], while some evidence suggest the opposite [30], we also assessed executive functioning performance. Particularly, we focused on behavioral inhibition, visual attention and task switching domains.

The Trail Making Test [31] assessed visual attention and task switching. In Part A, participants were instructed to connect randomly distributed numbers (from 1 to 25). In Part B of the Trail Making Test, participants were instructed to connect randomly distributed numbers (from 1 to 13) and letters (from A to M) in an alternating sequence (1-A-2-B-3-C …). Any participants who commit an error were instructed to correct it. The dependent measure of interest in the Trail Making Test was the time needed to finish the task in the Part B.

The Go/No-Go paradigm [32] involves a continuously presented series of stimuli composed of frequent “go” cues to which subjects respond as rapidly as possible and infrequent “no-go” cues to which subjects do not respond. The frequency of go cues (≥75%) creates a tendency to respond that must then be inhibited for no-go cues, thereby providing a measure of the ability to inhibit the response. In this study, participants heard several numbers, such as 4, 9, and 1. Every time participants heard a number, they should speak “yes”, unless when they heard the number 8 (no-go cue). Thus, the dependent measure of interest in this task was the number of errors.

### Social decision-making

Social decision-making experiments were conducted in anonymity. A trained team of psychologists conducted all interviews and experiments. In this study player 2 was a fictitious participant. Therefore, during the PD and UG the experimenters pretended to be communicating by cellphone with the fictitious player, who was supposed to be meeting with another experimenter in a different part of the city for control subjects, or in a different psychiatric unit for subjects with CUD.

### Prisoner’s dilemma (PD)

The PD experiment consists in a game in which two subjects who are unrelated are exposed to a fictional social decision-making situation [33]. The plot of the PD is that players are members of a criminal gang and they were arrested and imprisoned. Each player should imagine that she was actually a prisoner in solitary confinement, with no means of communicating with the other prisoner (fictitious player). The prosecutors were lacking sufficient evidence to convict the pair on the principal charge, but they had enough to convict both on a lesser charge. Therefore, the prosecutors offered each prisoner the option to testify that the other committed a crime, or to cooperate with the other by remaining silent. However, each decision would have resulted in the following outcomes associated with the length of prison sentence:

A. If both players betrayed each other, each of them would have served two years in prison;
B. If player 1 (research participant) betrayed player 2 (fictitious participant), but player 2 remained silent, player 1 would have been set free while player 2 would have served three years in prison (and vice versa);
C. If both players remained silent, both of them would have been served one year in prison;

According to game theory the dominant strategy for each participant would have been defection, because it offers a better payoff than cooperation (i.e. remain silent), regardless of the other player’s choice. From an economic perspective it is assumed that cooperation is an irrational choice, since it does not provide the highest amount of personal utility [34]. Thus, we also recorded the qualitative reason of why a given participant remained silent in the PD. These qualitative answers were grouped according to the decision’s motives, which allowed the creation of categories based on the combination of answers for further analysis. To do so, we performed thematic analysis on these answers, which is a method for identifying, analyzing, and reporting patterns (themes) within qualitative data. It minimally organizes and describes your data set in specific categories. In this sense, thematic analysis can be used to transform qualitative data into a quantitative form, and subject them to statistical analyses [35]. The unit of analysis tends to be more than a word or phrase, such as the motives of a given decision in the dilemma.

### Ultimatum game (UG)

The UG is based on a negotiation performed by two players (research participant and fictitious player). One player, the proposer, was endowed with a sum of chocolates (10 units of a popular chocolate covered wafer sticks ~ 6.5 g/each). The proposer was tasked with splitting it with another player, the responder. Once the proposer communicated their decision, the responder had the option to accept it or reject it. If the responder accepted, chocolates were divided according with the proposal; if the responder rejected the offer, both players received nothing. In this task, the players only had two chances to refuse or to accept the proposals, as well as to propose the chocolate division. In addition, each participant performed both positions of the game (proposer and responder) and their choices were recorded in each step.

The behavior of the fictitious player was always the same: as the female proposer, she offered 2 chocolates and wanted to keep 8 for himself. If the research participant (player 1) rejected the offer, the second proposal was as follows: 4 chocolates for player 1 and 6 chocolates for player 2. As the responder, the fictitious player always refused the first offer performed by player 1, and accepted any better offer that follows.

According to game theory, the divisions of chocolates that allowed one of the players to receive less than 4 chocolates are considered unfair offers [10].

### Data analysis

The Shapiro–Wilk test was used for the analysis of normality of data distribution for each variable. Descriptive statistics for sociodemographic, clinical, neuropsychological and social decision-making variables were conducted using chi-square tests or t-tests for independent samples. Multiple logistic regression models were used to analyze the effects of potential covariables (e.g. group, age, education and executive functioning) on the outcomes of social decision-making experiments. Using the enter method, Cox & Snell’s R-Square values were computed for each regression model.

Finally, the sample of the CUD group was subdivided based on the results of social decision-making experiments. Thus, two groups were generated: (1) Participants that opted to remain silent in the PD and also accepted the first unfair offer in the UG as responders (altered decision-making); and (2) Participants that opted to betray player 2 in the PD and also rejected the first “unfair” offer in the UG as responders (unaltered decision-making). Therefore, patterns of cocaine use were compared between these groups by using t-tests. All analyses were performed using the Statistical Package for the Social Sciences (SPSS) version 20.0 (SPSS Inc., Chicago, IL, USA), using two-sided tests and a significance level set at 0.05. The datasets used and/or analysed during the current study are available from the corresponding author on reasonable request.

## Results

### Descriptive statistics

Between groups comparisons on sociodemographic and clinical data are presented in Table 1. Participants of the CUD group were significantly older and had less years of formal education than participants of the control group. Moreover, participants of the CUD group reported significantly higher rates of substance use (alcohol, tobacco, cannabis and cocaine) and previous detentions, as well as increased frequency of psychiatric hospitalizations compared with controls. No significant group differences were observed for the average income, craving for chocolate and ethnicity.

**Table 1:** Between groups comparisons regarding sociodemographic and clinical data

	Controls (*n* = 55)	CUD (*n* = 129)	Statistics	*p*-value
Sociodemographic				
Age (years) – mean (SD)	26 (8.19)	30.43 (7.86)	t = 3.47	0.001
Ethnicity (white) **–** % (*n*)	53.4 (69)	66.6 (37)	χ2 = 3.53	0.316
Income (R$) – mean (SD)	2697.8 (2431.4)	2017.1 (2663.5)	t = 1.63	0.103
Years of formal education – mean (SD)	12.55 (4.28)	8.99 (3.71)	t = 5.71	0.001
Marital status (single) **–** % (*n*)	53.6 (30)	53.5 (69)	χ2 = 0.001	0.992
Previous detention **–** % (*n*)	1.8 (1)	20.2 (26)	χ2 = 10.57	0.001
Number of psychiatric hospitalizations – mean (SD)	0.05 (0.29)	1.42 (2.77)	t = 3.68	0.001
Craving for chocolate – mean (SD)	6.26 (2.87)	7.12 (2.90)	t = 1.84	0.066
Patterns of alcohol use				
Reported consumption **–** % (*n*)	87 (48)	96 (124)	χ2 = 8.42	0.015
Age of drug experimentation (year) – mean (SD)	15.19 (3.06)	14.25 (3.74)	t = 1.53	0.126
Patterns of tobacco use				
Reported consumption **–** % (*n*)	57 (31)	100 (129)	χ2 = 47.76	0.001
Age of drug experimentation (year) – mean (SD)	16.43 (5.35)	13.67 (3.09)	t = 3.75	0.009
Patterns of cannabis use				
Reported consumption **–** % (*n*)	23 (13)	72 (94)	χ2 = 88.83	0.001
Age of drug experimentation (year) – mean (SD)	16.23 (2.04)	17.70 (11.77)	t = 0.44	0.655
Drug use over time (days) – mean (SD)	169.82 (801)	2744.67 (2865)	t = 9.35	0.001
Patterns of cocaine use				
Reported consumption **–** % (*n*)	–	100 (129)	χ2 = 93.93	0.001
Age of drug experimentation (year) – mean (SD)	–	19.1 (5.77)		
Drug use over time (days) – mean (SD)	–	3571.62 (1931)		
Only snorted cocaine consume - % (*n*)		13 (10%)		
Only smoked cocaine consume - % (*n*)		7 (5%)		
Combined smoked and snorted cocaine consume - % (*n*)		109 (85%)		

Legend: Control – Healthy control females without a history of substance abuse; CUD – Females diagnosed with Cocaine Use Disorder

### Neuropsychological analyses

Between groups comparisons revealed that participants of the CUD group (mean = 126 s; standard deviation - SD = 67 s) took significantly longer to complete the Trial Making Test Part B (*t* = 4.92; *p* = 0.001), compared with controls (mean = 79 s; SD = 34 s). Moreover, participants of the CUD group (mean = 1.88; SD = 2.67) had significantly more errors in the Go/No-Go paradigm (*t* = 3.06; *p* = 0.003) than controls (mean = 0.73; SD = 1.32).

### Social decision-making experiments

In the PD, participants of the CUD group opted significantly more often to remain silent compared with controls (Table 2). To identify whether CUD would predict the outcome of the PD independently of the effects of other relevant variables, we performed two multiple logistic regression models. The first model included age, years of formal education and executive functioning measures as covariates, and it showed that the group effect remained significant (*R*^2^ = 0.128; Wald chi-square = 6.98; *p* = 0.008), even when controlling for the effects of age (Wald chi-square = 0.24; *p* = 0.620), education (Wald chi-square = 0.33; *p* = 0.562), performance in the Trail Making Test (Wald chi-square = 3.70; *p* = 0.062) and in the Go/No-Go paradigm (Wald chi-square = 0.29; *p* = 0.586). The second model accounted for the effects of the participant’s history of institutionalization, including as covariables the number of hospitalizations and if the participant had ever been in prison. This model also showed that the group effect on PD remained significant (*R*^2^ = 0.098; Wald chi-square = 11.88; *p* = 0.001), even when controlling for the effects of hospitalizations (Wald chi-square = 0.19; *p* = 0.661), and prison confinement (Wald chi-square = 1.06; *p* = 0.302).

**Table 2:** Between groups comparisons regarding social decision-making experiments

	Controls (n = 55)	CUD (n = 129)	Statistics	*p*-value
Prisoner’s Dilemma			χ2 = 17.05	0.001
Remain silent **–** % (*n*) [CI 95%]	33 (19) [21–46]	66 (86) [58–74]	–	–
Categories of motives (Remain silent)				
Lack of information **–** % (*n*) [CI 95%]	26 (5) [4–48]	51 (44) [40–62]	χ2 = 4.03	0.045
Injustice/Internal values **–** % (*n*) [CI 95%]	42 (8) [17–66]	36 (31) [26–46]	χ2 = 0.21	0.646
Strategy **–** % (*n*) [CI 95%]	31 (6) [8–54]	8 (7) [2–14]	χ2 = 7.73	0.005
Religiosity **–** % (*n*) [CI 95%]	0 (0) [0]	3 (3) [0–7]	χ2 = 0.69	0.406
Ultimatum Game				
1° propose **–** mean of offered chocolates (SD) [CI 95%]	4.26 (1.30) [3.91–4.61]	4.03 (1.31) [3.80–4.26]	*t* = 1.12	0.261
2° propose **–** mean of offered chocolates (SD) [CI 95%]	5.16 (1.59) [4.73–5.58]	5.19 (2.06) [4.83–5.55]	*t* = 0.10	0.915
1° response (acceptance of 2 chocolates) **–** % (*n*) [CI 95%]	39 (22) [26–52]	66 (86) [58–74]	χ2 = 12.04	0.001
2° response (acceptance of 4 chocolates) **–** % (*n*) [CI 95%]	73 (25) [57–89]	74 (32) [60–88]	χ2 = 0.10	0.930

Legend: Control – Healthy control females without a history of substance abuse; CUD – Females diagnosed with Cocaine Use Disorder. Propose – Analysis when participants proposed the chocolate division. Response - Analysis when participants were responders of the offers. CI 95% - Confidence Intervals

The reason that each participant reported for the choice of remaining silent in the dilemma was recorded, and subsequently 4 qualitative categories (lack of information, injustice/internal values, strategy, and religiosity) were generated based on these answers. Examples of answers were: (1) lack of information - “*I do not know who actually committed this crime*”, or “*I do not know if she did anything or not*”; (2) injustice/internal values – “*Because I would not lie, since I’m a good person*”, or “*Because I would not blame her, it would be unfair*”; (3) strategy – “*Because it’s my best chance*”, or “*If I betray her I can get a longer sentence*”; and (4) religiosity – “*Because I believe God is going to help me*”, or “*God is the one who decides what is right, not I*”.

The percentage of answers for each category is depicted in Table 2. Based on frequency comparisons, participants of the CUD group reported significantly more answers classified as lack of information compared with controls. Participants of the CUD group also reported significantly less answers related to the strategy category compared with controls. No group differences were observed for the categories of injustice and religiosity.

In the UG, no significant differences were observed when groups were analyzed as proposers, at both the first and second offers made by participants (Table 2). However, as responders, participants of the CUD group accepted significantly more often the first and unfair offer made by the fictitious player compared with controls, in which they received only 2 chocolates out of 10. Regarding the rate of acceptation in the second offer, no significant group differences were observed.

A multiple logistic regression model showed that the group effect detected in the acceptation rate of the first offer remained significant (*R*^2^ = 0.087; Wald chi-square = 7.26; *p* = 0.007), independently of the effects of age (Wald chi-square = 1.84; *p* = 0.175), education (Wald chi-square = 1.50; *p* = 0.220), performance in the Trail Making Test (Wald chi-square = 1.71; *p* = 0.190) and in the Go/No-Go paradigm (Wald chi-square = 0.01; *p* = 0.940).

### Subgroup analysis of participants with CUD

The group with CUD was subdivided based on the results of social decision-making experiments, showing that 51% (*n* = 66) of these participants were classified as having altered decision-making. Comparisons between the subgroups with and without altered decision-making were performed regarding the age of cocaine experimentation and cocaine use over time. While no differences were observed concerning the age of drug experimentation (*t* = 0.10; *p* = 0.990), participants of the subgroup with altered decision-making reported significantly more lifetime days of cocaine consumption (mean = 3948.14 days; SD = 1943) compared with the unaltered subgroup [(mean = 3151.32 days; SD = 1849) - (*t* = 2.02; *p* = 0.048)].

## Discussion

Here we examined the effects of CUD in social decision-making using the PD and the UG paradigms. The key findings are that females with CUD opted significantly more often to not defect in the PD, while in the UG they chose more often to accept the first and unfair offer as responders. Importantly, we have found that these effects were more pronounced in a subgroup of females with long-term history of cocaine use. Moreover, the results from logistic regression analyses suggested that associations between CUD and altered social decision-making are independent from demographic and neuropsychological variables, such as age, education level and regarding the performance in the Trail Making Test and in the Go/No-Go paradigm.

To our knowledge, this is the first study to investigate the performance of individuals with CUD in the PD. Specifically, we found that more than 60% of females who were cocaine dependents opted to remain silent and to not betray player 2 in order to avoid prison sentence. According to game theory, it is assumed that this pattern of “cooperation” in the one-shot PD is an irrational economic choice, since it does not provide the highest amount of personal utility, or in other words, the highest odds of receiving no sentence at all. However, it is worth noting that women in general present higher levels of cooperation in the PD when compared to men, even when controlling for socio-demographics factors [22]. In addition, evidence suggests that trust levels correlate with choices in the PD only for women and not for men [36]. In this case, it is suggested that CUD may potentiate the cooperation effect among women, and that future studies should consider gender differences when investigating CUD effects on PD.

Furthermore, we observed that participants of the CUD group reported significantly more answers classified as lack of information when explaining the reason for the choice of remaining silent, arguing that they did not know who had committed the crime, for instance. The judgment of who actually performed the illegal action in the PD is irrelevant for an economic perspective, given that participants should instead analyze the effects of each decision mostly based on prison sentence outcomes [37]. Indeed, we previously documented that females with CUD rarely used the available information to plan monetary riskless choices during non-social decision-making scenarios [15]. In this sense, it is possible to suggest that due to impairment in processing economic information during the evaluation of the dilemma options, females with CUD chose not to accuse the other player.

In addition to this economic decision-making context, there are a variety of elements that could have influenced the outcomes of the PD, including personality [38] and environmental factors [39]. In a broader view, one could argue that when females with CUD did not have proper information about the role of the other player in the fictitious criminal scenario, they rather remain silent, particularly because who was the author of the crime was a key element for solving the dilemma. Instead of rationally analyzing prison sentence, these women may have considered that testifying that the other committed the crime requires much more evidence and facts, suggesting they were more sensitive to the criminal plot of the PD than controls. In fact, a history of legal issues is associated with more mutual cooperation and reciprocity in the PD, given that cooperation rates among female inmates exceed the rate of cooperation among female students [40]. It is suggested that the social structure among prisoners is such that you survive better if you cooperate, while defecting could be a negative option because inmates could be more likely to run into fellow participants afterward [40]. Moreover, evidence suggests that real-life prisoners are not inherently more selfish than individuals without any history of problems with justice [41], except for prisoners with antisocial personality disorder [42]. Taking into account that females with CUD reported significantly higher rates of legal detentions and psychiatric hospitalizations in our study, such history of institutionalization may have influenced their ability to judge one’s own and others’ behavior in the specific scenario of the PD, even though the logistic regression model that accounted for such effect did not show statistical significance. It is still possible that this may leaded them to a more cooperative behavior, particularly when they lacked information about who actually committed the felony.

Furthermore, our data showed that females with CUD exhibited higher acceptation rate of unfair offers in the UG when playing as responders. Overall, we interpret this finding as showing they were willing to accept any amount of chocolate regardless of whether this was a fair/unfair proposal. In this sense, two chocolates were better than none, which would have been the outcome if they rejected the first offer. This idea fits well with previous evidence showing altered subjective sensitive to reward in cocaine abusers. For instance, in an experiment where cocaine abusers and healthy controls had to choose between different amounts of monetary reward, more than half of the cocaine abusers rated $10 as equally subjectively valuable as $1000 [43]. This low sensitivity in individuals with CUD would diminish the distinction process between stimuli of different gradations, and this would implicate in an all-or-nothing pattern of decision during the UG, particularly when performing the position of responder in this game. This is also supported by findings showing that CUD is associated with dampened reward response to social interaction [44], suggesting an imbalance between the exaggerated value given to drugs and the devalued appeal for natural reinforces, such as chocolates and social stimulus [12].

Neurobiological findings suggest hypo-activation in the insula and the anterior cingulate regions of the brain among individuals with CUD while performing the UG, which has been interpreted as reflecting lower emotional processing of unfairness and less sensitivity to social violations [11]. Thus, our data provide tentative evidence that during drug abstinence, females with CUD may present reduced processing of unfairness, but also compromised subjective sensitive to reward in general.

This study has certain limitations. First, the PD and the UG were tested in their short-versions (e.g. one-shot, two-shot), which did not allow us to investigate the effects of CUD repeatedly upon these games, as well as regarding reinforcement learning [45]. Second, the CUD group was older, had lower education and had more institutionalizations when compared to the control group. Although these group differences may influence at some level the outcomes of social decision-making, such background profile of chronic cocaine users can be hardly detached from a patient. For instance, it has been shown that CUD is associated with low levels of education [46], and with detentions and admissions in inpatient mental health units [47]. Thus, the idea of the control group of non-substance abusers was to include a normative sample for the purpose of comparison, although we did not match case-patients with controls. However, we controlled the effect of these possibly confounding variables throughout the study by performing logistic regressions. Third, females with CUD were restrained in a psychiatric unit for drug detoxification during the experiments, while controls participants were not. Thus, such environmental factor associated with a history of institutionalization might have influenced the results as aforementioned discussed.

However, the study also has some important strengths. First, based on our logistic regression models, we provided further evidence that social decision-making is a cognitive process apart, at least at some level, from other brain processes such as general executive functions [28]. Thus, although previous studies have documented deficits in executive functions (e.g. inhibition, task-switching, etc.) among cocaine dependents, it appears that there is not an uniform frontal-lobe effect of chronic cocaine use on cognition. In fact, dual-processing models of higher order cognition introduced the notion of “cold” executive functions, comprising planning, working memory, and behavioral inhibition, and “hot” executive functions, including social cognition, empathy, and emotion regulation [48]. Second, this is one of the few studies attempting to integrate distinct game theory paradigms to investigate social decision-making using a larger sample of individuals with CUD compared with previous attempts [1, 2, 11]. This helped to identify differences between individuals with and without cocaine dependence that previous studies were not able to reveal, mostly because of small sample sizes [11].

## Conclusions

In conclusion, social decision-making in adult females is influenced by CUD. The association of altered decision-making with cocaine dependence was detected in both social paradigms utilized, resulting in more cooperative behavior in the PD and higher acceptance rate of unfair offers in the UG. However, the chain of events leading to these outcomes is still unknown. Despite that, this study emphasizes that social decision-making is an important aspect closely related to clinical outcomes in CUD, given that long-term cocaine use was associated with an even more altered performance in social game tasks. This is particularly relevant given the fact that many cocaine abusers present impairments in social functioning. Thus, social game paradigms such as the UG and the PD can provide important and complementary assessments of social functioning and decision-making, allowing a better comprehension of how patients behave when they are engaging in social contexts [1]. Further studies should focus on investigating these associations to shed light on the putative biopsychosocial factors underlying the observed effects.

## Data Availability

The datasets used and/or analysed during the current study are available from the corresponding author on reasonable request.

## Abbreviations

CUD: Cocaine use disorder
PD: The Prisoner’s Dilemma
UG: The Ultimatum Game
